# Understanding Bioluminescence in Dinoflagellates—How Far Have We Come?

**DOI:** 10.3390/microorganisms1010003

**Published:** 2013-09-05

**Authors:** Martha Valiadi, Debora Iglesias-Rodriguez

**Affiliations:** 1 Department of Evolutionary Ecology, Max Planck Institute for Evolutionary Biology, August-Thienemann-Strasse, Plӧn 24306, Germany; 2 Department of Ecology, Evolution and Marine Biology, University of California Santa Barbara, Santa Barbara, CA 93106, USA; E-Mail: debora.iglesias-rodriguez@lifesci.ucsb.edu

**Keywords:** diel rhythms, functional diversity, luciferase, luciferin binding protein, gene, evolution, ecology, scintillon, predation defense

## Abstract

Some dinoflagellates possess the remarkable genetic, biochemical, and cellular machinery to produce bioluminescence. Bioluminescent species appear to be ubiquitous in surface waters globally and include numerous cosmopolitan and harmful taxa. Nevertheless, bioluminescence remains an enigmatic topic in biology, particularly with regard to the organisms’ lifestyle. In this paper, we review the literature on the cellular mechanisms, molecular evolution, diversity, and ecology of bioluminescence in dinoflagellates, highlighting significant discoveries of the last quarter of a century. We identify significant gaps in our knowledge and conflicting information and propose some important research questions that need to be addressed to advance this research field.

## 1. Introduction

The biological production of light or bioluminescence is a widespread phenomenon in nature. Bioluminescence is particularly predominant in the marine environment, in members of planktonic bacteria and protozoa, many invertebrates, and vertebrates with specialized light producing organs that harbor symbiotic bioluminescent bacteria (reviewed by [1]). Marine bioluminescence is mainly blue and, therefore, tuned to the wavelengths that travel furthest through the water. Light is produced by a chemical reaction in the presence of oxygen involving a substrate termed “luciferin” and the enzyme “luciferase”. The production of these molecules and their mobilization within the cell for the production of light is thought to incur a high energetic cost. Despite that, in a remarkable example of convergent evolution, bioluminescence is estimated to have evolved independently at least forty times [1,2,3]. This has led to the chemistries of bioluminescent reactions being as diverse and unrelated as the organisms that produce them [2,3,4].

Dinoflagellates are the main eukaryotic protists that are capable of producing light [1,5]. Within this group, bioluminescence is present in a number of ecologically important species, many of which formblooms [6,7]. Indeed, dinoflagellates are responsible for most of the bioluminescence observed in the surface ocean [8]. Particularly when their populations are dense, disturbance of the water during the night causes bright blue bioluminescent displays that have been reported since at least 500 BC [9] and are known to occur globally [10]. Considering the ecological importance of dinoflagellates, e.g., HABs, primary production, extensive grazing, symbiosis, parasitism (reviewed by [11]), and their prominence as producers of bioluminescence in the ocean, this fascinating phenomenon has received little attention. In this paper, we review recent progress in this topic, highlighting the major findings reported in the last quarter of a century. We also discuss findings that have led us to question key paradigms in the field or those that have revealed major gaps in our knowledge and we suggest future research directions to further our understanding of this fascinating phenomenon.

## 2. Light Production in the Cell

As with all bioluminescence systems, the one present in dinoflagellates is unique from both a cellular and molecular perspective. The production of light occurs in organelles termed scintillons [12], which contain the luciferin substrate, the luciferase enzyme (LCF) and, in some species, a luciferin binding protein (LBP) [13,14,15,16]. Scintillons are dense vesicles approximately 0.5–0.9 µm in diameter [14,17] and which, during the hours of darkness, are abundantin the periphery of the cell [18,19]. Light is primarily produced in response to mechanical stimulation due to shear stress [20], for example upon contact with grazers or by breaking waves [21].

**Figure 1:**
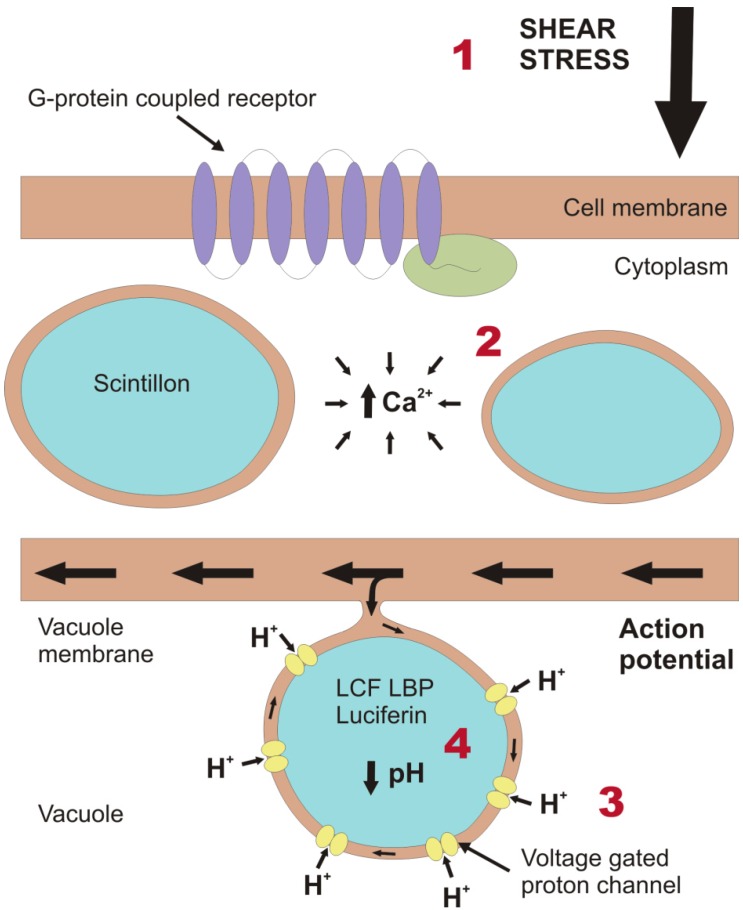
Schematic representation of part of a dinoflagellate cell, depicting the cellular processes that take place to generate a bioluminescence flash. (1) Initially, a force is exerted on the outer cell membrane. Transduction involves the activation of a G-protein coupled receptor [22] (where G stands for GTP-binding), which typically consists of a receptor (purple) comprising seven transmembrane domains and the G-protein (green) on the inner side of the cell membrane. Subsequent to transmission of the mechanical signal from the outside to the inside of the cell, the signal must be translated to an electrical signal on the vacuole membrane. (2) This is achieved by increasing the concentration of Ca^2+^ in the cytoplasm, mainly from sources within the cell but also from the external medium [24]. This causes the vacuole membrane to depolarize and generate an action potential. In this illustration, one of the scintillons protrudes into the vacuole and is connected to the vacuole membrane. Thus, the action potential can travel across the membrane of the scintillon. (3) This results in the activation of voltage gated proton channels, which we here assume to be concentrated on the membrane of the scintillon rather than on the remaining vacuole membrane. (4) The influx of protons from the acidic vacuole rapidly lowers the pH of the scintillons and activates the bioluminescence chemistry. While some of the protons may be sequestered by the production of water as a by-product of bioluminescence, the fate of the rest of these protons and the mechanism by which the scintillons return to the original pH values are unknown.

A cascade of cellular processes mediates bioluminescence, from sensing a stimulus to the production of light (Figure 1). The chemical reaction to produce light is pH dependent, requiring the acidification of scintillons. For this reason, some scintillons protrude into the acidic vacuole. The shear stress exerted on the cell membrane triggers a mechanotransduction pathway to generate an action potential across the vacuole membrane and the associated scintillons. Chen *et al*. [22] provided evidence that this cascade involves the activation of GTP-binding protein coupled receptors in the plasma membrane. It is, however, unknown at which stage these receptors are activated. They areunlikely to be primary sensors as the fastest G-protein signaling pathway known to date has a longer latency time than the whole transduction process in dinoflagellates [23]. Experiments by von Dassow and Latz [24] conducted using various ionic inhibitors showed that, after sensing the mechanical disturbance, the next step of the process is mediated by an increase in cytosolic Ca^2+^, mainly by release from intracellular stores and partially from extracellular sources. The action potential produced by these processes leads to an influx of protons from the acidic vacuole into the scintillons, decreasing their internal pH from ~8 to ~6 [25].

The acidification of the scintillons had long been hypothesized to be mediated by a voltage gated proton channel, and, indeed, Smith *et al*. [26] cloned and expressed a gene for such a unique channel that is highly proton selective, pH sensitive and capable of inward proton flux. Although this work was carried out on the non-bioluminescent dinoflagellate *Karlodinium veneficum* because of the availability of sequence data, this channel may be present in other dinoflagellates. When acidification of the scintillons is achieved it causes structural changes in LCF leading to its active conformation and making the luciferin binding sites available [27]. In species that contain LBP, this protein binds the luciferin at neutral to alkaline pH, protecting it from autoxidation, and releases it by means of conformational change below pH 7, at which LCF also becomes active. Luciferin is oxidized by LCF to form oxyluciferin, which results in the emission of photons (reviewed by [28,29]) in the form of a brief flash of blue light (with an approximate wavelength of 475 nm). The exact details of the chemical process and the intermediate molecule that produces the light during the oxidation process are unknown. In general, the mechanotransduction pathway is remarkable as the transfer of the signal from the cell membrane to the vacuole membrane and the mobilization of all the components needed to produce a bioluminescent flash takes place in just 20 ms.

## 3. Molecular Composition and Evolution of the Bioluminescence System

### 3.1. Luciferases and Luciferin Binding Proteins

The complex evolution of dinoflagellate bioluminescence systems has been revealed through the study of the dinoflagellate luciferase gene (*lcf*) which has been fully sequenced in seven genetically closely related photosynthetic dinoflagellates of the Gonyaulacales genera *Alexandrium*, *Lingulodinium*, *Protoceratium*, and *Pyrocystis* [30,31] and in the heterotrophic species *Noctiluca scintillans* [32]. As with nearly all dinoflagellate protein coding genes, multiple non-identical copies in tandem arrangement are present in each organism. In addition, there may be distinct gene variants encoding different isoforms of the protein and therefore rather than “genes” they can be considered gene families.

#### 3.1.1. Luciferase Genes of Photosynthetic Species

The luciferase gene was first cloned and characterized from the dinoflagellate species *Lingulodinium polyedrum* (=*Gonyaulax polyedra*) [33,34] and then later found to exist in the same organization in six other species [30]. It is composed of three tandemly repeated domains (D1, D2, and D3) each consisting of a central region that is highly conserved at the amino acid level among domains and species, and encodes a catalytically active site. This region is flanked by more variable *N*- and *C*-terminal regions, the roles of which are to control the pH response and activity of the enzyme, respectively [35,36]. Indeed, four histidine residues in the *N*-terminal regions of each domain are thought to induce the pH-mediated conformational change that exposes the otherwise folded catalytic sites [27,36]. An *N*-terminal gene region, which shows similarities to glutathione-*S*-transferase at the amino acid level, precedes the three catalytic domains in all studied species but its function is unknown [30,31].

Despite the similar organization of *lcf* in the photosynthetic species studied so far, there are species differences in the distribution of synonymous substitution rates across the gene open reading frame. While these are evenly distributed in the *lcf* of two *Pyrocystis* and two *Alexandrium* species, they are almost nil in the central domain regions which code for the enzyme catalytic sites in *L. polyedrum* and *Protoceratium reticulatum* [30,37]. The intergenic regions of *lcf* also vary considerably between species [38]. The significance of these differences among genes is unclear, especially if one assumes that the evolutionary constraints on bioluminescence are likely to be the same on the *lcf* of all these organisms. Some of these differences may, however, relate to different regulatory elements acting in these organisms, particularly with regard to the diel cycle of bioluminescence [30,37] (explained in Section 5.1). In addition, three types of *lcf* are known to exist in *Pyrocystis lunula* and one of them represents the only *lcf* known to contain an intron [37].

An intriguing feature of the three domain *lcf* genes of dinoflagellates is their evolution. Sequence comparisons of individual domains from different species have shown that corresponding domains of *lcf* are more similar in different organisms (e.g., D1 of *A. tamarense* and D1 of *P. reticulatum*) than are different domains within each organism (e.g., D1 and D2 of *A. tamarense*) [30]. This is likely to reflect an ancient triplication of a *lcf* domain that was then carried forward during the evolution of the different photosynthetic species [30]. However, the group of photosynthetic dinoflagellates that has been studied comprises very closely related species and it is therefore likely that much more variation exists, even within photosynthetic species. For instance, a highly divergent *lcf* exists within *Ceratium digitatum* [7], which is a representative of a cosmopolitan open ocean genus that has been found to dominate the bioluminescent field in the North Atlantic during late summer and autumn [39].

#### 3.1.2. Luciferin Binding Protein Genes of Photosynthetic Species

The luciferin binding protein performs the important role of binding luciferin at physiological pH, but it has received a lot less attention than luciferase. A reason for this might be that it does not exist in all bioluminescent dinoflagellates and therefore has not been considered as an essential component of the bioluminescence system. The presence of the LBP gene (*lbp*) is well known in *L. polyedrum* [16,40], *N. scintillans* [32] and it has also been found in the transcriptomes of four *Alexandrium* species [41,42,43,44]. In fact, the only genus confirmed to lack expressed LBP is *Pyrocystis*, whose protein extracts have been screened by a universal antibody for LBP [15,16]. Indeed, how many of the bioluminescent dinoflagellate species utilize LBP in their bioluminescence system is currently unknown.

The only species in which *lbp* has been well characterized is *L. polyedrum.* Its *lbp* occurs as a gene family with two distinct variants with approximately 86% identity [45,46], each composed of four repeat domains with little similarity between them, unlike the homologous domains *lcf* [32]. Both variants have been found to be expressed at equal levels [45]. The *lcf* and *lbp* of *L. polyedrum* show similarities such as a homologous *N*-terminal gene region [31] and a lack of introns and typical transcription promoters [46], which agrees well with their translational control through the light-dark cycle [47]. The physical structure of LBP has not yet been characterized and so it is unclear how exactly it binds luciferin, but it is thought to exist as a dimer that binds one luciferin molecule and releases it by conformational change below pH 7 [47].

Recent research on dinoflagellate transcriptomes has revealed that *lbp* mRNAs dominate the transcript pool of two *Alexandrium* species [41,42,48]. Likewise, LBP has been found to be very abundant (1%) in the *L. polyedrum* proteome [47]. Transcriptome studies of *A. catenella* [42,48] and *L. polyedrum* [49] suggest that LBP is produced at a higher rate than LCF. Likewise, the concentration of LCF is estimated to be approximately 100 times lower than the value reported for LBP [50]. This could be explained if LCF can be recycled in the bioluminescent reaction while LBP is proportional to the amount of luciferin present. These results clearly indicate that, in organisms where it is present, LBP is a core part of the bioluminescence system and therefore more studies into the identity and origin of this protein are needed. This information may also explain how *P. lunula* bioluminescence operates without LBP, which is counterintuitive for a gene that seems to be central to the bioluminescence system in other species. Elucidating the crystal structure of LBP will aid in understanding how two proteins (LCF and LBP) of which corresponding gene sequences show no similarities have co-evolved to bind the same substrate and synchronize their activities to the same pH range.

#### 3.1.3. Insights from the Bioluminescence Gene of *Noctiluca scintillans*

**Figure 2:**
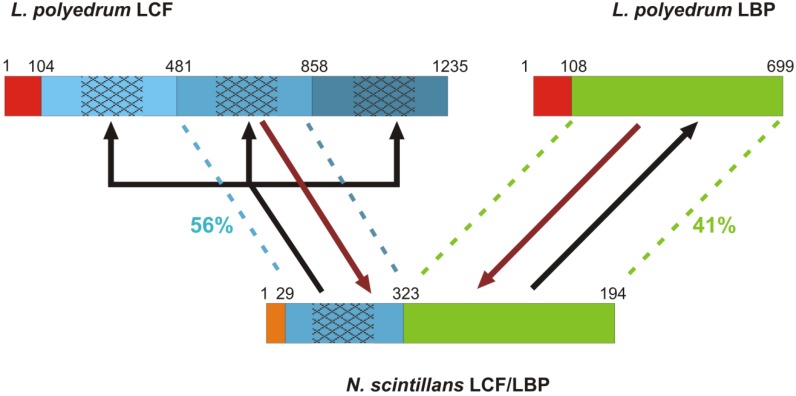
A schematic representation of the different LCF and LBP structures labeled with amino acid positions and the two scenarios for their evolution [30,32]. *Lingulodinium polyedrum* LCF represents the photosynthetic dinoflagellates in general [30]. The three non-identical repeat domains of *L. polyedrum* LCF (different shades of blue) are each 377 amino acids long with 150 amino acids in the central regions that encode the catalytic sites (patterned) and retain higher sequence similarity. The single LCF domain of *N. scintillans* is most similar to the second domain of *L. polyedrum* LCF. The LBP domain in *N. scintillans* is structurally equivalent to that in *L. polyedrum*, both consisting of four repeat domains. In both organisms, LCF is preceded by an *N*-terminal gene region which is similar in the LCF and LBP of *L. polyedrum* (red) but not in *N. scintillans* (orange). Two potential scenarios of how these gene structures arose are shown. In the gene fission scenario (black line), the *N. scintillans* gene split and the LCF domain underwent successive duplications. In the gene fusion scenario (brown lines), the second domain of LCF was excised (e.g., by splicing) and fused with LBP in *N. scintillans*.

The sequence of the bioluminescence gene of *N. scintillans* obtained by Liu and Hastings [32] was the first representative of a heterotrophic dinoflagellate and outside the order Gonyaulacales, the focus of all previous work. This proved to be a significant milestone in understanding the extreme diversity of *lcf* in dinoflagellates. The *lcf* in *N. scintillans* is unique as it consists of only one domain which is shorter than that of photosynthetic species and is attached to *lbp* as a single hybrid gene (Figure 2). Its *N*-terminal gene region lacks any sequence similarity and is much shorter than that of photosynthetic species. Additionally, the *lcf* domain is shorter by 60 amino acids at the *N*-terminal than the *lcf* domains of photosynthetic species and is thus missing three of the four histidines that are thought to be responsible for pH regulation. Nevertheless, the pH regulation remains. Despite these major structural differences, both the functionally relevant central part of the *lcf* domain and *lbp* domain retain sequence similarity at the amino acid level to *L. polyedrum*. The origin of this hybrid gene in *N. scintillans* has been difficult to interpret [32] and its significance is controversial [51,52]. The triplication of *lcf* domains in the seven studied photosynthetic dinoflagellates is thought to have occurred in their common ancestor before their divergence [30]. Therefore, if *N. scintillans* is a primitive dinoflagellate as suggested by many phylogenies, the *lcf*/*lbp* could have undergone fission giving rise to the separate genes in photosynthetic species [32]. However, if it is a more recently derived lineage, the separate *lcf* and *lbp* could have fused in this species, with reduction of *lcf* to a single domain [32]. The order of these events is important in understanding the evolution of these proteins in relation to different selection pressures that may require differential regulation pathways of these genes. The most recent phylogenetic analysis of *N. scintillans* and other noctilucoids based on ribosomal genes, suggests that it is indeed a primitive dinoflagellate species [53]. However, a phylogeny based on a combined analysis of ribosomal and protein-coding genes has questioned this conclusion [54]. In this case, *N. scintillans* may have acquired its bioluminescence gene by horizontal transfer and subsequent modification from another dinoflagellate, which is consistent with bioluminescence being absent in other noctilucoid dinoflagellates. Regardless of which scenario is true, the triplication of the separate *lcf* in photosynthetic species has been suggested to confer an advantage by tripling the catalytic capacity that can be achieved within the scintillons without a tripling of their protein content that would increase their osmotic pressure [33]. It is therefore important to ascertain whether evolution is selecting for higher bioluminescence capacity within each scintillon. The answers to these questions will come with complete sequencing of bioluminescence genes from other species, especially including representatives of taxonomic groups that are distinct from the ones already sequenced, for example, *Ceratium* and *Protoperidinium* spp. The key questions that remain to be answered are: (1) is there an *lcf* with two domains? (2) which species contain *lbp*? (3) when did *lbp* become part of or was lost from *lcf*?

### 3.2. The Elusive Luciferin

The structure of the luciferin molecule has only been characterized in *P. lunula* due to the relative ease of extraction because cells lack a theca and because they contain approximately 100 times more luciferin that *L. polyedrum* [16]. The structure of *P. lunula* luciferin was fully characterized by Nakamura *et al.* [55] as a linear tetrapyrrole with similarity to chlorophyll a. This molecule exhibits a characteristic blue fluorescence (475 nm) when irradiated with UV light, a feature that is also used to visualize scintillons in cells by epifluorescence microscopy. However, the fluorescence of the molecule ceases when it has been oxidized either by LCF or by air. A study with amino acid tracers has confirmed the intracellular production of luciferin in *P. lunula* [56] and Topalov and Kishi [57] suggested that luciferin is a product of chlorophyll degradation by means of photooxidation. 

It is generally assumed that all dinoflagellate luciferins are the same as in *P. lunula* because its luciferin has been shown to cross react with the LCF of all other bioluminescent dinoflagellates species [16,58]. This is also the case with the euphasiid shrimp (krill) *Euphasia superba*, the LCF of which can also cross-react with *P. lunula* luciferin. Indeed, the luciferin of *E. superba* is very similar to that of *P. lunula* but it is more closely related to chlorophyll c [57], presumably obtained from its prey. A similar hypothesis for the dietary acquisition of chlorophyll for luciferin production has been put forward for the heterotroph *N. scintillans* [32]. However, a recent study on another heterotrophic dinoflagellate, *Protoperidinium crassipes*, showed that bioluminescence intensity persisted after the cells were cultured for one year on rice flour, hence, luciferin could not have been synthesized from chlorophyll but rather must have been produced endogenously [59]. It is important to note that while luciferins and LCF cross-react even between phylogenetically very distant organisms such as dinoflagellates and krill, their LCF exhibits completely different characteristics. The LCF of euphasiids has not been cloned but its molecular weight is 600 kDa [60] in contrast to 135 kDa of the LCF in photosynthetic dinoflagellates [30,33]. In addition, euphausiid LCF exhibits much slower kinetics than dinoflagellate LCF. Therefore, it seems that bioluminescence systems exhibit some degree of flexibility in the reactants used to produce light. Additionally, the hypothesis that the origin of luciferin is photo-oxidated chlorophyll [57] is only plausible for *P. lunula*, which maintains its luciferin throughout the light-dark cycle. Fluorescent luciferin only appears in *L. polyedrum* at the onset of the dark-phase [13] thus, its formation cannot be explained by the photo-oxidation mechanism. Therefore, it is likely that more than one mechanism is responsible for the production of luciferin, even in photosynthetic species with closely related LCFs.

## 4. Diversity of Bioluminescent Species

Several bioluminescent species are cosmopolitan in both coastal and open ocean regions and include important heterotrophs (e.g., *Noctiluca* and *Protoperidinium*) and toxic (e.g., *Alexandrium*), or generally harmful species (e.g., *Noctiluca*, *Lingulodinium*, and *Ceratium*). Although bioluminescent species have been reported to occur in 17 dinoflagellate genera (compiled by [6]) recent work in our lab [7] clarified some ambiguities that stemmed from inaccurate field observations and confusion from inherent variation in bioluminescence among clonal strains. Key genera such as *Ceratium* and *Protoperidinium* have not been studied in enough detail to enable conclusive reporting of their bioluminescent members, but as a guide, Table 1 indicates which genera are dominated by bioluminescent species and those in which bioluminescence is less predominant.

A lot of effort has been put into characterizing bioluminescent dinoflagellate species. An obvious reason for this is that it aids the classification of cells identified by microscopy into bioluminescent and non-bioluminescent groups. One purpose of this information can be to determine the extent to which various taxa contribute to the total bioluminescence elicited from a body of water (e.g., [39,61]). A further purpose can be to investigate the seasonality of bioluminescent dinoflagellate populations in time series, especially in relation to the total dinoflagellate populations. Another important use of this information is in investigating potential evolutionary similarities of bioluminescent species, or how bioluminescence may have been acquired and lost among and within different dinoflagellate lineages.

**Table 1:** Genera with reported bioluminescent species from Marcinko *et al.* [62], Poupin *et al.* [6], and Valiadi *et al.* [7]. The numbers of species within each genus is according to the taxonomically accepted species listed in Algae Base [63]. The question mark indicates that the report is anecdotal and not confirmed.

Order	Genus	No. of reported	Total no. of
Family		BL species	species in genus
Gonyaulacales			
Ceratiaceae	*(Neo)ceratium*	4	77
Goniodomaceae	*Alexandrium*	7	31
	*Pyrodinium*	1	1
Cladopixidaceae?	*Peridiniella*	1	3
Ceratocoryaceae	*Ceratocorys*	1	11
Gonyaulaceae	*Gonyaulax*	11	72
	*Lingulodinium*	1	2
Pyrocystaceae	*Pyrocystis*	4	16
Pyrophacaceae	*Fragilidium*	4	5
	*Pyrophacus*	1	4
Gymnodiniales			
Gymnodiniaceae	*Polykrikos*	2	5
Noctilucales			
Noctilucaceae	*Noctiluca*	1	1
Peridiniales			
Proroperidiniaceae	*Protoperidinium*	31	295

The two largest genera of bioluminescent dinoflagellates both in terms of size and range, *Ceratium* and *Protoperidinium*, contain a very low proportion of bioluminescent representatives (Table 1). Presumably, this is because they are notoriously difficult to maintain in culture and, therefore, their taxonomic classification has not been studied in depth, especially with molecular markers. Indeed, dinoflagellate species complexes that were considered as one genus have often been re-classified after extensive investigations as separate genera, for example, the genus *Gonyaulax* from which *Lingulodinium*, *Alexandrium*, and *Protoceratium* were defined. Tracing the lineage specific evolutionary pathways of bioluminescence will greatly benefit from detailed studies of species/strains belonging to these two ecologically important genera. This will be important as both *Ceratium* and *Protoperidinium* are highly represented in natural bioluminescent dinoflagellate populations [39]. Finally, although the ability to produce light is exhibited by phylogenetically disparate dinoflagellate taxa, it is dominated by the Gonyaulacales, suggesting that either, both the genes and cellular machinery for bioluminescence were born within this group, or they were acquired from a less conspicuous ancestor.

## 5. Factors Affecting the Intensity of Bioluminescence Emissions

The measurement of the bioluminescence signature in a dinoflagellate culture or a single cell reveals a complex signal whose features vary from species to species and cell to cell, time of day, and the physiological status of the cell. This section describes some of the most common factors that affect the characteristics of bioluminescence signatures in different organisms with their unique life histories.

### 5.1. Diurnal Rhythms of Bioluminescence

Most bioluminescent dinoflagellates display a diurnal rhythm in bioluminescence intensity, being much brighter in the night than in the day, when it is almost negligible. The two main mechanisms underlying this variation are the regulation of bioluminescence at the molecular and cellular level by a circadian clock and/or photoinhibition of bioluminescence in the day.

In photosynthetic dinoflagellates, bioluminescence exhibits a diurnal rhythm controlled by an endogenous circadian clock. This makes bioluminescence almost undetectable in the day and brightest at night. The regulation of the bioluminescence circadian rhythms varies significantly in the two dinoflagellate species (*L. polyedrum* and *P. lunula*) that have been extensively studied (reviewed in depth by [64]). In *L. polyedrum*, the quantity of LCF and LBP is translationally regulated, while mRNA levels of both genes remain stable throughout the light-dark cycle [15,65,66]. It has been suggested that this process is mediated by a repressor protein which binds to the 3′ untranslated region of the *lcf* mRNA [67], although this has been disputed [68]. The LCF, LBP, luciferin, and the scintillons themselves are destroyed at dawn and then begin to be resynthesized at dusk; they peak in quantity approximately four hours into the night when they reach amounts that are 10 times higher than in the day [13,18,50,69]. In contrast, *P. lunula* regulates its bioluminescence in a completely different way. The number of scintillons and their luciferin and LCF content, as well as *lcf* mRNA, do not vary [15,37]. Instead, the scintillons are relocated interchangeably with the chloroplasts to modulate bioluminescence intensity, placing the scintillons at the periphery of the cell during the night but near the center of the cell during the day to prevent their stimulation [19].

Besides a circadian rhythm, bioluminescence intensity is diminished in the day by photoinhibition. This is likely to occur in all dinoflagellates as it has been extensively documented in both photosynthetic and heterotrophic species [70,71,72,73,74,75,76]. Only two exceptions have been reported, one being *N. scintillans* [76] and the other being a natural population of *Protoperidinium antarcticum* [77], which is in disagreement with observations in other *Protoperidinium* species [73,76]. The majority of bioluminescent species are most sensitive to blue light, which reduces their sensitivity to mechanical stimulation, irrespective of the amount of LCF or luciferin contained in the cell [70,73,74,78]. Neither the mechanism by which photoinhibition takes place, nor the identity of the receptor of the inhibiting light, has been found. Nevertheless, photoinhibition of bioluminescence is considered an important evolutionary adaptation to tune bioluminescence to the times when it is visible and minimize unnecessary energy expenditure.

The molecular regulation of circadian rhythms has received a lot more attention than photoinhibition. Photoinhibition is rather more universal in bioluminescent dinoflagellates than are circadian rhythms, since many heterotrophic species such as *Protoperidinium* do not show circadian regulation of their bioluminescence but do show photoinhibition [76,79]. The importance of the photoinhibition of bioluminescence in nature has been shown in *in situ* mixed populations containing both photosynthetic and heterotrophic dinoflagellates [61,79,80], whereas circadian rhythms may be important when the bioluminescent population is dominated by photosynthetic species (e.g., [61,81]). Nevertheless, photoinhibition is ecologically more relevant than circadian rhythms because natural dinoflagellate populations are inevitably exposed to natural day-night cycles in the euphotic zone.

A much less investigated phenomenon that also exhibits circadian control is spontaneous bioluminescence. This has been recorded in *L. polyedrum* [82], *P. lunula* [81,83], *Pyrodinium bahamense* [81], and *Ceratocorys horrida* [84]. *Lingulodinium polyedrum* emits spontaneous flashes at the beginning of the dark phase and a continuous glow at the end of the dark phase. *Pyrocystis lunula* and *P. bahamense* emit spontaneous flashes during the whole dark phase but do not generate significant glow. A constant background low-level light emission has been seen in all three species during dark phase. *Ceratocorys horrida* emits spontaneous flashes throughout the dark phase and a glow at the end. Spontaneous flashing and glowing and background low level emissions are likely to represent biochemical and cellular processes that could shed light on the endogenous (*i.e*., not externally stimulated) function of bioluminescence.

### 5.2. Species-Specific Bioluminescence Signatures

There seem to be considerable differences in flash characteristics among species. For example, the duration of a flash varies significantly, between 80 ms in *N. scintillans* [85], 130–150 ms in *L. polyedrum* [84] and 500 ms in *Pyrocystis fusiformis* [86]. In addition, the brightness of the emitted light differs considerably among species, ranging from 10^7^ photons per cell in *Gonyaulax excavata* (synonym *Alexandrium tamarense*) [87] to 10^9^ photons per cell in *Pyrocystis noctiluca* [88]. These differences could be due to a number of reasons. An important factor is thought to be cell size, with larger cells emitting more light [89], because the number of scintillons a cell can contain is proportional to its size. For example, cells of *L. polyedrum* in the dark phase contain on average 320 scintillons per cell [18], while the larger *Noctiluca scintillans* contains 100,000–200,000 scintillons per cell [90,91]. The amount of available luciferin can also vary by 100-fold [16] and it is probably related to scintillon number. Furthermore, the number of scintillons stimulated in one flash has been found to be as few as 5% in *N. scintillans* [85]. Likewise, only a small fraction of the molecules involved, around 15% in *P. fusiformis* [86], are used up in one flash. In addition, the sensitivity of each species to mechanical stimulation is variable [92]. It is also important to note that in cultures it is inherently difficult to obtain ecologically-relevant measurements of species-specific flash intensity as culture conditions tend to diminish bioluminescence over time [93,94]. For example, not all cells within a monoclonal culture contain scintillons and those that do may have varying numbers [95]. Intraspecific variation in bioluminescence presence/absence and intensity has also been observed in isolates from a single natural population of *P. lunula* [96]. Therefore, the characteristics of bioluminescence produced by each species, or even every cell, can differ significantly because of several simultaneous endogenous controls.

### 5.3. Physiological State

The intensity of bioluminescence can be affected by the physiological status of the cells and by environmental factors. Bioluminescence may diminish when heterotrophic cells are starved [73,76,97] and when cultures of photosynthetic species become nutrient depleted [70,98]. Furthermore, combined effects are known in *Protoperidinium depressum* in which the bioluminescence system became even more sensitive to photoinhibition after a period of starvation [73]. However, Latz and Jeong [99] found that bioluminescence correlated with feeding frequency rather than growth rate in *P. divergens* and *P. crassipes* and that cells continued to invest energy in bioluminescence despite being unable to grow. In photosynthetic species, the response of bioluminescence intensity to limitation by different nutrients has not been explicitly assessed. However, a recent study of the transcriptome of *Alexandrium fundyense* showed that the expression of *lbp* was higher in phosphate limitation than in nitrate limitation [41].

Bioluminescence can also be photoenhanced, whereby the light emitted in the dark is proportional to the total amount of light received in the preceding light phase. This has been reported in *L. polyedrum* cultures [75], in laboratory cultures and natural populations of *Ceratium fusus* [100], and in natural populations dominated by *Gonyaulax* species [61]. However, the biochemical and physiological processes leading to this phenomenon have not been explored. Specifically, it is unclear whether the changes in bioluminescence intensity are due to changes in the sensitivity of the cells to mechanical stimulation or to differences in the production of substrates for bioluminescence under different irradiance regimes. Investigating these questions may shed light on the reciprocal interactions between bioluminescence and other photo-regulated metabolic pathways.

## 6. The Function of Bioluminescence in Dinoflagellates

The functions of marine bioluminescence have mainly been studied in deep-sea megafauna and in bacteria, where it plays a range of important roles, including oxygen defense, predator avoidance, camouflage and courtship (reviewed by [1,2]), in various bioluminescent organisms. However, the function of bioluminescence in dinoflagellates has been less extensively assessed and the theoretical concepts that have been put forward are only supported by limited experimental evidence. 

**Figure 3:**
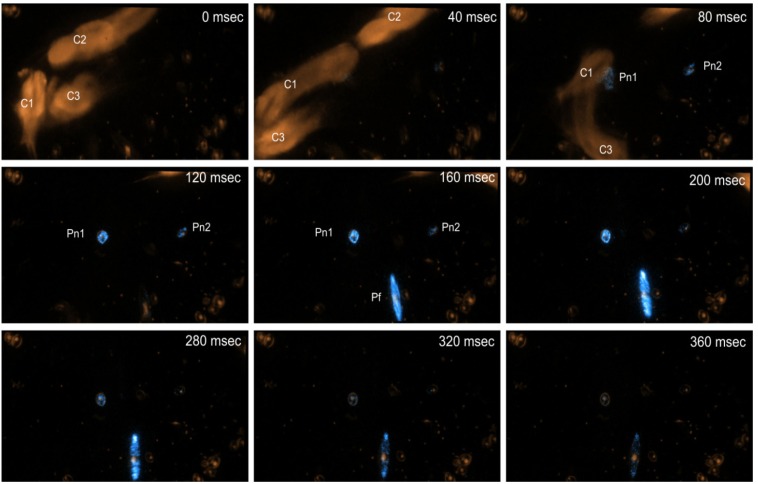
Image sequence from a video of bioluminescent dinoflagellates flashing in response to stimulation by copepods. The time from the beginning of the image sequence is indicated on each frame. Three copepods (C1–C3) collide and rapidly swim away from each other. During these fast swimming bursts, the dinoflagellates are stimulated to bioluminesce, although it is not possible to discern whether this is due to the copepod swimming current or to direct contact. Bioluminescence is seen to develop and diminish in the scintillons of two cells of *Pyrocystis noctiluca* (Pn) and a *Pyrocystis fusiformis* (Pf), which is pulled into the field of view at 160 ms by the copepod swimming current. The video was filmed by Ammonite Films at the National Oceanography Centre—Southampton. Filming was done under an inverted microscope at 10× magnification. The outline was captured using infrared light and bioluminescence was captured by a blue sensitive camera.

Dinoflagellate bioluminescence is proposed to act as defense against predation (recently reviewed by Marcinko *et al*. [62]). It is well known that shear stimulates dinoflagellate bioluminescence [20,92], for example, due to proximity or contact with zooplankton. An example of this interaction is shown in Figure 3. Early studies by Esaias and Curl [101] and White [102] showed that the higher the bioluminescence intensity of the dinoflagellate *Gonyaulax* sp. (by using cells at different stages of the dark phase) the fewer the cells consumed by the copepod genera *Acartia* and *Calanus*, most likely due to the disruption of the copepods’ swimming and feeding behavior by bioluminescent flashes [103]. Photophobic responses of marine zooplankton to artificial flashes of light have been reported in several studies [103,104,105] and the authors have suggested that bioluminescence could confer an evolutionary advantage to dinoflagellates by reducing predation pressure directly through startling and repelling their predators.

A widely accepted theory is that bioluminescence protects dinoflagellates indirectly by acting as a “burglar alarm” [106]: when a flash is stimulated by contact with a grazer, it will attract a higher-level predator that will then consume the grazer. This theory received a lot of attention in the early 1990s with several studies seeking to demonstrate it experimentally. In general, these experiments have compared predation rates on zooplankton by higher-level predators in the presence of bioluminescent dinoflagellate cells whose natural bioluminescence intensity could be varied by using cells at different phases of their light-dark period. The findings of the two key studies in the literature agreed despite using very different experimental communities, one containing the dinoflagellate *L. polyedrum*, the copepod *Tigriopus japonicus* and three spine sticklebacks [107], and the other containing the dinoflagellate *P. fusiformis*, the mysid *Holmesimysiscostata* and the midshipman fish *Porichthys notatus* [108]*.* In both studies, the authors postulated that even if the dinoflagellate which flashes (*i.e*., the bioluminescent genotype) dies, the population as a whole will still benefit because blooms form asexually and therefore the organisms are genetically highly similar. This would indeed be the case in asexually reproducing bloom-forming populations composed of genetically similar individuals. However, there is significant intraspecific variation in bioluminescence intensity in natural populations [96] and bioluminescence does not occur only in bloom-forming dinoflagellates.

Although all these studies have shown important elements of bioluminescence effects on two trophic levels and claim that dinoflagellates can benefit from the reduced number of grazers, they present two significant experimental caveats. First, dinoflagellate densities at the beginning and end of each experiment have not been reported [107,108] mainly because the adverse effect of bacterial contamination incurred by the introduction of a large animal could not be assessed [107]. Second and foremost, none of these experiments have used organisms that co-occur in nature. For example, Mesinger and Case [108] used mysids and fish from Santa Barbara, California, but *P. fusiformis* does not occur in this region. Rather, in this experiment, the use of *L. polyedrum*, which is endemic to Southern California’s coast, would have been more ecologically relevant. In addition, even if organisms from the same area were used, they may be separated seasonally and therefore may not encounter each other naturally. It is entirely plausible that this swimming behavior of zooplankton in response to dinoflagellate bioluminescence may lead to zooplankton exclusion zones from areas in the water column with high densities of bioluminescent dinoflagellates. This could even benefit other organisms that may be mixed within the dinoflagellate population, including other dinoflagellates.

The limited understanding of the ecological significance of bioluminescence in dinoflagellates partly stems from the lack of *in situ* studies on these organisms. Planktonic bioluminescence has been explored in the coastal and open oceans only by measuring total bioluminescence of both dinoflagellates and zooplankton using bathyphotometers. There have been two major field programs of this type, one in the Sargasso Sea (Biowatt—[80,109,110]) and one in the North Atlantic (Marine Light Mixed Layers—[39,111]), that have directly investigated relationships between stimulated bioluminescence and the bioluminescent organisms present with the overarching aim of predicting bioluminescence in the water column (recently reviewed by [62]). As optical bioluminescence measurements are in discriminatory to the array of organisms that produce light and because there are temporal changes in the contributions of dinoflagellates and zooplankton to the total photon budget, these studies have not been able to show a consistent relationship of bioluminescence to any environmental factors. Furthermore, dinoflagellate bioluminescence signatures can be affected by several parameters as outlined in Section 5 and are thus difficult to use in estimating the abundance of bioluminescent dinoflagellate cells. Therefore, in order to explore the distribution and composition of bioluminescent dinoflagellate populations in the ocean, an alternative approach to optical measurements is needed. A better characterization of bioluminescent species or their molecular detection in the field may be helpful in this direction [7,112].

A metabolic or cell physiological role for bioluminescence has never been explored. A surprising finding was made by Erdner and Anderson [41] in a study of the transcriptome of *Alexandrium fundyense* which showed that the expression of *lbp* was higher under phosphate limitation than under nitrate limitation, therefore showing the different relationships of bioluminescence to these two key metabolic processes. The importance of bioluminescence, particularly of LBP, to the life of the cell has been documented in several dinoflagellate transcriptome studies. Jaeckisch *et al.* [44] reported that *lbp* was the eighth most expressed gene in *Alexandrium ostenfeldii*. Uribe *et al.* [42] found that *lbp* was the most highly expressed gene in *A. catenella* representing 3% of the total expressed sequence tags, whereas genes involved in core functions like cell cycle and metabolism were expressed at lower levels. Similar results were reported by Toulza *et al.* [48] using a different strain of *A. catenella*. A main difference between these studies was that *lcf* expression was ranked either second [42] or sixteenth [48]. Despite this discrepancy, which may be due to differences in the employed sequencing technology, time of sampling, or due to culturing effects (e.g., mutations leading to loss of expression from some *lbp* copies), both studies agree that *lcf* expression is high. As mentioned previously, the high expression level of a bioluminescence protein has also been reported for *L. polyedrum* [47]. Despite these striking findings, not a single metabolic physiological study on bioluminescence has been reported.

A recent hypothesis for the original function of bioluminescence has been put forward by Wilson and Hastings [29]. In their “oxygen defense” hypothesis they argue that bioluminescence systems evolved in response to low oxygen levels during the time between the great oxidation event (~2 billion years ago), when photosynthesis evolved, and the Cambrian explosion (500–550 million years ago). Wilson and Hastings suggest that because all bioluminescence systems consume oxygen, they detoxified organisms from oxygen, with light being a simple by-product. Bioluminescence would have then acquired a different functional role when antioxidant pathways, such as superoxide dismutases and catalases, became widespread with increasing oxygen levels. Their hypothesis is mainly based on the bioluminescence systems of bacteria and fireflies but it is indeed plausible for the other bioluminescent organisms as well. In dinoflagellates, this scenario would require that the early chemistry was not pH regulated since lowering the pH to “activate” LCF and LBP can currently only take place in the scintillons. These are not thought to be independent vesicles but would have rather evolved later to tune bioluminescence to specific responses like mechanical stimulation. Such a scenario would also mean that every trace of bioluminescence has been eliminated from basal dinoflagellate lineages. At present there is not enough evidence to evaluate this hypothesis. Progress in both *lcf* sequencing and characterization of luciferin in more organisms is needed to explore the likelihood of the “oxygen defense” hypothesis in dinoflagellates. However, regardless of whether or not this hypothesis holds true, bioluminescence in dinoflagellates will serve an alternative function in the present day as it is not known to provide them with any advantage in fighting oxidative stress.

## 7. Conclusions and Future Directions

Bioluminescence is an innovation in the life of many dinoflagellates species with substantial visual, ecological and cellular effects and is, therefore, an integral part of their lifestyle. Much progress has been made in recent years towards understanding this process, particularly regarding the cellular mechanisms, circadian rhythms and the molecular evolution of bioluminescence in dinoflagellates. However, in this review we have highlighted the infant status of our knowledge of the evolution, mechanism and controls on bioluminescence and raised several new questions that need to be investigated.

The cellular mechanisms of bioluminescence and the molecular evolution of *lcf* and *lbp* are fairly well characterized. The greatest highlights of the last 25 years have been the sequencing of *lcf* from several species, especially *N. scintillans*, the characterization of *P. lunula* luciferin and significant progress in our understanding of the cellular components and mechanotransduction pathway that induce the production of light. The collation of data on bioluminescent species and the first ecological experiments involving bioluminescent dinoflagellates have also resulted in significant insight into the topic. However, important questions about the identity of luciferin and the evolutionary processes that have led to the current configuration of *lcf* and *lbp* have arisen in recent years. One research priority is to determine the structure and origin of luciferin in bioluminescent dinoflagellate species other than *P. lunula*, preferably a heterotroph cultured without photosynthetic prey and a species that is known to contain LBP such as *L. polyedrum*. This will capture any potential variation in the structure and origin of luciferin in distinct groups and provide important insights into how the bioluminescence system may be linked to other metabolic functions. Further characterization of LBP, especially its structure and distribution within dinoflagellates is needed to understand why this protein is so abundant in many bioluminescent species and how it has co-evolved with the rest of the bioluminescence system. Full sequences of *lcf* from a variety of organisms are needed for two reasons: to enable tracing the evolutionary origin of bioluminescence and to assess how the composition of the bioluminescence system has changed over time in relation to differential regulatory requirements or selection pressures on each gene and its variants. For example, *L. polyedrum* is ~30 times smaller and contains at least 300 times fewer scintillons than *N. scintillans* [18,90,91] and yet, its bioluminescence intensity is only ~200 times less [76,93]. This is consistent with the proposal by Liu and Hastings [30] that the triplicate structure of LCF allows cells to triple the catalytic capacity within the scintillons without increasing the osmotic pressure. It also means that smaller cells can achieve the highest possible bioluminescence intensity relative to their scintillon carrying capacity, suggesting that selection acts towards higher bioluminescence in these populations.

Bioluminescence is difficult to place in an ecological context because its function is not fully understood. Indeed, the function of bioluminescence has been investigated much less than any other topic in the field. The predator defense hypotheses have not yet been sufficiently investigated. The “oxygen defense”, if true, may explain how bioluminescence initially arose and evolved before it developed an effect on another group of organisms that could be selected for. However, it does not imply that bioluminescence has the same function in the modern ocean. Also, the environmental regulation of bioluminescence remains an open question. A good place to start would be to investigate changes in bioluminescence, gene/protein expression and luciferin content in response to changing environmental conditions, such as nutrient availability, that are frequently encountered in nature.

As with all functional traits of an organism, it is more realistic to investigate the function of bioluminescence in its natural context. While the complexity of natural ecosystems makes this task difficult, observations in nature can reveal the true selection pressures and ecological dynamics of bioluminescence. Blooms that frequently occur in coastal ecosystems can provide a natural laboratory for such studies and desired measurements can be easily incorporated into existing time series monitoring programs (e.g., those for monitoring HABs). Measurements of population structure in relation to bioluminescence at the molecular and phenotypic level, followed by linking to observations of co-occurring organisms, particularly non-bioluminescent dinoflagellates, zooplankton and potential prey, nutrients levels and irradiance levels would be a starting point to investigate the function and ecology of dinoflagellate bioluminescence.

Since ecology and evolution are inseparable, integrated studies of gene evolution and regulation with new clues on the environmental factors and ecological interactions that regulate bioluminescence will help us understand the role of this incredible functional innovation in the life of dinoflagellates.
